# THE VILLAGE MODEL, WHAT’S NEXT?

**DOI:** 10.1093/geroni/igz038.1523

**Published:** 2019-11-08

**Authors:** Carrie Graham, Andrew Scharlach

**Affiliations:** University of California, Berkeley, California, United States

## Abstract

Researchers at UC Berkeley will present some key findings of their research on Villages spanning the last decade. First, results of a longitudinal study of operational Village organizations in the US (conducted in 2009, 2012, and 2016) reveals that the Village model has expanded and developed over time, with some changes in organizational structure. A national survey of Village members (N=2000) shows that Village remain homogeneous, and impact different types of members in different ways, with older, more frail members perceiving more quality of life benefit, while younger, healthier members perceive more benefits in the areas of social engagement. Finally, two studies looking at Village retention/participation show that issues such as lack of diversity, focus on social engagement can be barriers to inclusion, retention and ultimately, scalability of the model.

